# Cerebrocortical Beta Activity in Overweight Humans Responds to Insulin Detemir

**DOI:** 10.1371/journal.pone.0001196

**Published:** 2007-11-21

**Authors:** Otto Tschritter, Anita M. Hennige, Hubert Preissl, Katarina Porubska, Silke A. Schäfer, Werner Lutzenberger, Fausto Machicao, Niels Birbaumer, Andreas Fritsche, Hans-Ulrich Häring

**Affiliations:** 1 Department of Internal Medicine IV, University of Tübingen, Tübingen, Germany; 2 Institute of Medical Psychology and Behavioral Neurobiology, University of Tübingen, Tübingen, Germany; 3 Department of Obstetrics and Gynecology, College of Medicine, University of Arkansas for Medical Sciences, Little Rock, Arkansas, United States of America; 4 Department of Neuro-Ophthalmology, University Eye Hospital, Tübingen, Germany; 5 National Institutes of Health (NIH), National Institute of Neurological Disorders and Stroke (NINDS), Human Cortical Physiology, Bethesda, Maryland, United States of America; University of California at Los Angeles, United States of America

## Abstract

**Background:**

Insulin stimulates cerebrocortical beta and theta activity in lean humans. This effect is reduced in obese individuals indicating cerebrocortical insulin resistance. In the present study we tested whether insulin detemir is a suitable tool to restore the cerebral insulin response in overweight humans. This approach is based on studies in mice where we could recently demonstrate increased brain tissue concentrations of insulin and increased insulin signaling in the hypothalamus and cerebral cortex following peripheral injection of insulin detemir.

**Methodology/Principal Findings:**

We studied activity of the cerebral cortex using magnetoencephalography in 12 lean and 34 overweight non-diabetic humans during a 2-step hyperinsulinemic euglycemic clamp (each step 90 min) with human insulin (HI) and saline infusion (S). In 10 overweight subjects we additionally performed the euglycemic clamp with insulin detemir (D). While human insulin administration did not change cerebrocortical activity relative to saline (p = 0.90) in overweight subjects, beta activity increased during D administration (basal 59±3 fT, 1^st^ step 62±3 fT, 2^nd^ step 66±5, p = 0.001, D vs. HI). As under this condition glucose infusion rates were lower with D than with HI (p = 0.003), it can be excluded that the cerebral effect is the consequence of a systemic effect. The total effect of insulin detemir on beta activity was not different from the human insulin effect in lean subjects (p = 0.78).

**Conclusions/Significance:**

Despite cerebrocortical resistance to human insulin, insulin detemir increased beta activity in overweight human subjects similarly as human insulin in lean subjects. These data suggest that the decreased cerebral beta activity response in overweight subjects can be restored by insulin detemir.

## Introduction

The role of insulin signaling to the brain in normal physiology and pathophysiology is so far only incompletely understood. The majority of data on insulin action in the brain was obtained in animal models, only few studies characterize insulin action in human brain.

Peripherally injected insulin crosses the blood-brain barrier [1], [2] and contributes to the regulation of food intake and energy homeostasis [3]. Insulin signaling in the brain serves as a feed-back signal from the periphery to the brain to reduce appetite [4], [5]. Furthermore, insulin receptors in the brain seem to be involved in pathogenic mechanisms leading to a type 2 diabetes-like phenotype. The selective neuronal knockout of the insulin receptor resulted in an obese and insulin resistant phenotype [6]. Activation of insulin receptors in the central nervous system has been shown to be essential for appropriate suppression of endogenous glucose production during hyperinsulinemia in mouse models [7]–[9]. Furthermore, insulin has been found to be associated with cognitive function in rodents [10]. In humans, intranasal administration of insulin, an application which raises insulin levels in the cerebrospinal fluid and selectively stimulates the brain [11], improves memory function [12]. Moreover, reduced insulin signaling is a pathogenic factor of Alzheimer's disease [13], [14] and might be associated with loss of cognitive and memory function.

In a previous study we measured insulin effects on neuronal activity of the human cerebral cortex using magnetoencephalography (MEG) during a 2-step hyperinsulinemic euglycemic clamp [15]. In this study we observed that in obese individuals the brain appears to be resistant to stimulation of beta- and theta-activity by insulin. In subjects with a body-mass-index (BMI) of approximately 30 kg/m^2^, and elevated body fat content, a physiologic dose of insulin did not exert a detectable effect on this parameter of brain activity. These results raised the question whether therapeutic tools exist to restore the cerebral insulin response in overweight humans.

Modern insulin therapy regimens in type 1 and type 2 diabetes aim to mimick the patterns of physiologic insulin secretion. For this purpose, long- and short-acting insulin analogues have been designed in recent years. Insulin detemir is a long-acting insulin analogue and its delay of action is achieved by acylation of the B-chain with myristic acid and reversible albumin binding [16], [17]. The pharmacokinetics differ from human insulin and may therefore cause tissue-specific effects.

It has been shown that the transport of insulin across the blood-brain barrier is reduced in dogs with obesity induced by a high-fat diet [18]. Therefore, insulin resistance of the brain is at least in part a consequence of reduced availability of insulin and may be overcome by insulin analogues with altered pharmacokinetics and tissue-selectivity for the brain. In a recent animal study we found that peripheral injection of insulin detemir results in higher brain tissue concentrations of this insulin analogue compared to human insulin in the presence of similar peripheral effects. Subsequently, insulin receptor signaling in the hypothalamus and cerebral cortex was enhanced when insulin detemir was injected i.v., and electroencephalography recordings in these animals displayed a different stimulation of the cortical activity compared to human insulin [19].

Based on these findings in mice, we hypothesized that insulin detemir might restore the decreased cerebral insulin response in overweight human subjects. Therefore, we designed a 2-step hyperinsulinemic euglycemic clamp with i.v. infusion of insulin detemir or human insulin and simultaneous MEG recording. To ensure that potential cerebral effects are not a spill over from peripheral insulin effects, we applied clamp conditions which avoided overstimulation of peripheral tissues with insulin detemir.

## Methods

### Subjects

Here we selected 10 overweight or slightly obese subjects who were otherwise healthy and normal glucose tolerant according to WHO criteria. As indicator of overweight, a percentage body fat above the sex-specific normal range (male >19%, female >28%) was taken. Body fat was measured by bioelectrical impedance analysis using a BIA101A analyzer (RJL systems, Clinton Twp., MI 48035 USA). Differentiation of overweight from normal body weight by percent body fat content resulted in inclusion of two female subjects with a BMI slightly below 25 kg/m^2^ but increased body fat. Severe obesity (BMI >40 kg/m^2^) and/or psychiatric disorders represented exclusion criteria. No subject took any medication except for hormone substitution (like thyroid hormones).

In these 10 subjects we studied the effect of insulin detemir and human insulin on cerebrocortical activity. The effect of insulin detemir on cerebrocortical function was then compared to the human insulin effect measured in 12 lean and 34 obese non-diabetic subjects. The subject characteristics of all three groups are shown in table 1. Informed written consent was obtained from all subjects prior to the study. The study protocol was approved by the Ethics Committee of the Medical Faculty at the Eberhard-Karls-University in Tübingen, Germany.

**Table 1:** Subjects characteristics.

	Lean	Overweight	Overweight, insulin detemir**	
	Mean±SEM	Mean±SEM	Mean±SEM	Range
N	12	34	10	-
Sex (M/F)	4/8	19/15	5/5	-
Age (years)	29±2	36±2	30±2	[23 … 42]
Weight (kg)	62±3	88±2	88±6	[67 … 119]
BMI (kg/m^2^)	22±1	29±3	29±1	[23 … 36]
Percent body fat (%)	19±2	31±1	30±2	[21 … 42]
Percent body fat, females (%)	24±1	37±1	35±4	[30 … 42]
Percent body fat, males (%)	10±1	26±5	25±4	[21 … 29]
Waist-hip-ratio	0.80±0.02	0.91±0.01	0.88±0.04	[0.66 … 0.98]
Fasting plasma glucose (mmol/L)	4.7±0.1	5.0±0.1	5.2±0.1	[4.6 … 5.7]
2 Hr plasma glucose (mmol/L)*	5.6±0.4	6.4±0.2	5.8±0.4	[4.1 … 7.7]
Fasting plasma insulin (pmol/L)	37±3	61±6	52±10	[24 … 108]
2 Hr plasma insulin (pmol/L)*	195±56	468±65	360±99	[69 … 989]

* Oral glucose tolerance test;

** subset of the overweight group

### Oral glucose tolerance test

After a 10-hour overnight fast the subjects ingested a solution containing 75 g of dextrose and venous blood samples were obtained at 0, 30, 60, 90 and 120 minutes for determination of plasma glucose and plasma insulin.

### Experimental design

**Figure 1:**
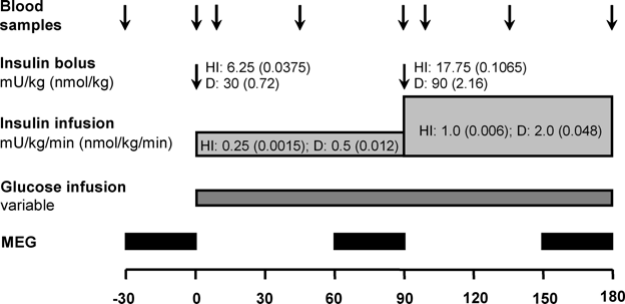
The 2-step hyperinsulinemic euglycemic clamp with human insulin or insulin detemir. Human insulin (HI) and insulin detemir (D) were applied as a 2-step infusion. Each infusion step was primed with a bolus. Blood glucose was monitored every 5–10 min between minute 0 and minute 180, and a variable glucose infusion was titrated to maintain euglycemia (blood glucose 5 mmol/l). Cerebrocortical activity was measured with magnetoencephalography (MEG) during the baseline period and during the last 30 minutes of each insulin infusion step. In the control experiment, a comparable volume of saline solution (S) was infused instead of HI, D and glucose. The MEG measurement, glucose monitoring and blood sampling were performed analogously to the clamp experiments.

The human insulin experiment and the saline experiment with simultaneous MEG recordings have already been established and used in a previous study [15]. In addition, we designed a 2-step hyperinsulinemic euglycemic clamp with i.v. infusion of insulin detemir. In this clamp a similar or even slightly lower effect on peripheral glucose metabolism had to be achieved and the time-profile of the plasma insulin concentrations had to be mimicked. We first assessed the dose of insulin detemir in consideration of previous studies on insulin detemir action during i.v. infusion in humans and pharmacological data on insulin detemir kinetics. After a pilot study to test the clamp protocol, 10 subjects participated in these three experiments (insulin detemir [D], human insulin [HI] and saline[S]) on three different days approximately one week apart. The order of the experiments was randomized and all participants were blinded as to whether human insulin, insulin detemir or saline was infused. An approximately 30-minute MEG block was performed in the baseline period and at the end of each step of the clamp. Further details of the clamp procedures and the bolus and infusion doses of human insulin and insulin detemir are given in figure 1. To avoid substantial adhesion of insulin detemir and human insulin to plastic materials like the infusion lines, the insulin solutions were prepared using 48 mL NaCl 0.9% and 2 mL of the subjects' own blood.

### Magnetoencephalography (MEG)

We chose MEG parameters that permitted sensitive assessment of both spontaneous cortical activity and stimulated cortical activity (discrimination between two sound qualities, auditory mismatch negativity). Data were recorded in a continuous mode (sampling rate 312.5 Hertz [Hz]) starting with eyes open and closed (counterbalanced over sessions and subjects) for 1.5 minutes each for analysis of spontaneous cortical activity, followed by the auditory mismatch experiment. Auditory mismatch negativity is independent of alertness or attention and is considered to be a robust parameter of preconscious cortical information processing [20], [21]. While the auditory mismatch experiment was different in some details, the analysis of spontaneous cortical activity was performed as previously described [15], including a correction for multiple comparisons in the different frequency bands by a randomization approach [22].

### Analytical procedures

Plasma glucose was determined using the glucose-oxidase method (YSI, Yellow Springs Instruments, Yellow Springs, CO, USA). Blood glucose was determined by a HemoCue blood glucose photometer using the glucose dehyrogenase method (HemoCue AB, Aengelholm, Sweden). Plasma insulin levels were determined by microparticle enzyme immunoassay (Abbott Laboratories, Tokyo, Japan).

### Statistical analysis

MEG data was analyzed by a repeated measure ANOVA containing the condition factor (SUBSTANCE, e.g. human insulin and insulin detemir) and the repeated measure factor level (baseline, 1^st^ step and 2^nd^ step of insulin infusion). To visualize the relative changes under human insulin and insulin detemir MEG data of the insulin/detemir experiment were divided by data of the saline experiment. MEG parameters were calculated using SPSS 12.0 (SPSS, Chicago, IL) incorporating Greenhouse-Geisser correction. For analysis of metabolic data the software package JMP 4.0 (SAS Institute, Cary, NC) was used. Non-normally distributed variables were logarithmically transformed, when necessary. Correlations were calculated using least square regression analysis. A p-value of <0.05 was considered to indicate statistical significance.

## Results

### Kinetics of human insulin and insulin detemir concentrations in 10 overweight subjects participating in the human insulin, insulin detemir and saline experiment

**Figure 2:**
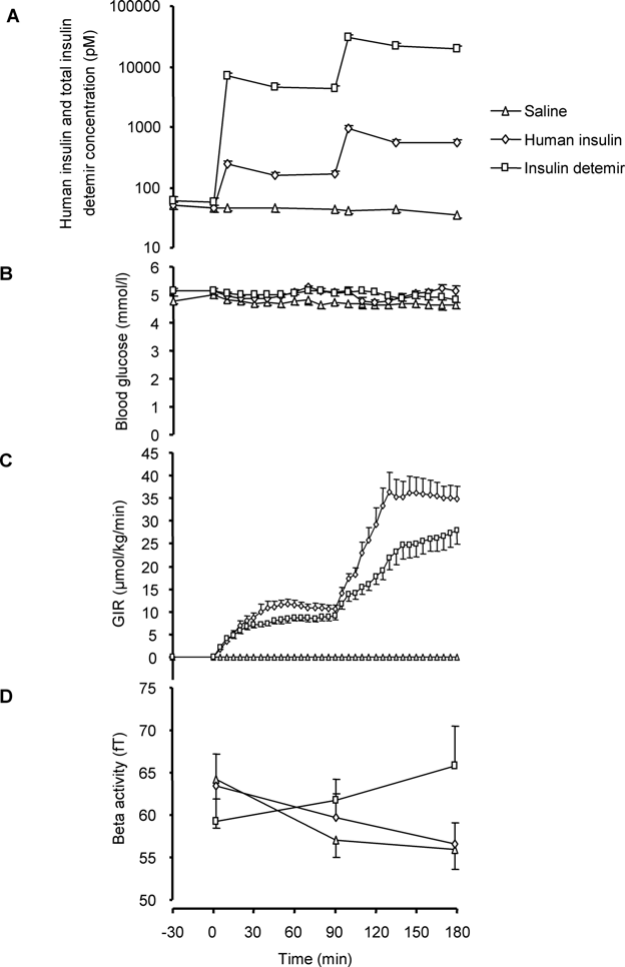
Metabolic parameters and beta activity during infusion of human insulin, insulin detemir and saline in 10 overweight individuals. A: Plasma human insulin and total serum insulin detemir concentrations. Similar profiles of plasma/serum levels (Mean±SE) of human insulin and insulin detemir were achieved with both insulins. Approximately 98% of serum insulin detemir is bound to albumin. Therefore, total insulin detemir concentrations are substantially higher than human insulin concentrations at corresponding time points. Human insulin was determined by microparticle enzyme immunoassay (Abbott Laboratories, Tokyo, Japan) and insulin detemir by a modified radioimmunoassay (Capio Diagnostik, Copenhagen, Denmark). B: Blood glucose concentrations. Blood glucose was measured twice at baseline and every 5–10 minutes during the infusion of human insulin, insulin detemir or saline. Blood glucose concentrations were not different between the experiments at baseline (all p>0.1) and did not change significantly during the experiments (all p>0.2). C: Glucose infusion rates. In the saline experiment no glucose was infused. Despite much higher insulin detemir concentrations, the glucose infusion rate was lower in the detemir experiment, indicating a lower peripheral effect (1^st^ step: insulin 11±1 µmol kg^−1^ min^−1^, insulin detemir 9±1 µmol kg^−1^ min^−1^, p = 0.01; 2^nd^ step: insulin 36±3 µmol kg^−1^ min^−1^, insulin detemir 26±3 µmol kg^−1^ min^−1^, p = 0.003), as intended by the experimental protocol. D: Changes in beta activity during the experiments. During human insulin (HI) and saline (S) infusion, beta activity decreased slightly (p = 0.70, HI vs. S). During insulin detemir (D) infusion beta activity increased compared to HI (p = 0.001, repeated measures ANOVA, adjusted for multiple testing in all frequency bands).

Figure 1 shows the design of the clamp experiments. During the saline experiment (S), plasma insulin levels slightly decreased from basal 49±7 pM to 1^st^ step 48±6 pM and 2^nd^ step 40±5 pM (p = 0.04) (Figure 2A).

In the human insulin experiment (HI) and the insulin detemir experiment (D) basal plasma insulin concentration were not different from S (HI 48±6 pM, p = 0.91; D 57±10 pM, p = 0.16). During both clamps, plasma insulin and total serum insulin detemir concentrations displayed similar time profiles (Figure 2A). In each step the peak value after the priming bolus was approximately 55% higher than the concentrations observed during infusion of human insulin and insulin detemir, respectively. However, due to the albumin binding [16] total serum concentrations of insulin detemir were substantially higher (approximately 40-fold) at any time point than the corresponding plasma insulin concentrations (Figure 2A).

### Peripheral metabolic effects of human insulin and insulin detemir in 10 subjects

Blood glucose concentrations were not different at baseline (S 4.8±0.4 mM, HI 5.0±0.3 mM, D 5.0±0.5 mM, all p>0.1) and did not change significantly during the experiments (all p>0.2) (Figure 2B).

Though the total insulin detemir concentrations were higher than the human insulin concentrations, the glucose infusion rate required to maintain euglycemia was lower in the insulin detemir experiment (Figure 2C). The lower glucose infusion rate indicates that despite these high insulin detemir concentrations, no overstimulation of peripheral glucose metabolism occurred.

### Cerebrocortical measures of human insulin and insulin detemir action in 10 overweight subjects

As we screened all frequency bands of spontaneous cerebrocortical activity for differences between HI, S and D, we used a randomization approach to adjust for multiple testing as previously described [22]. As evoked responses we analyzed auditory mismatch negativity. Between the saline and the human insulin experiment spontaneous (Figure 2D) and evoked cerebrocortical activity (data not shown) were not different, confirming our previous finding that in overweight and obese subjects the insulin response of the brain is reduced [15]. However, during the insulin detemir experiment, beta activity increased from 59±3 fT at basal to 62±3 fT in the 1^st^ step and 66±5 fT in the 2^nd^ step (p = 0.001, detemir vs. human insulin) (Figure 2D).

### Comparison of the insulin detemir effect on beta activity with the human insulin effect in 12 lean and 34 obese subjects

**Figure 3:**
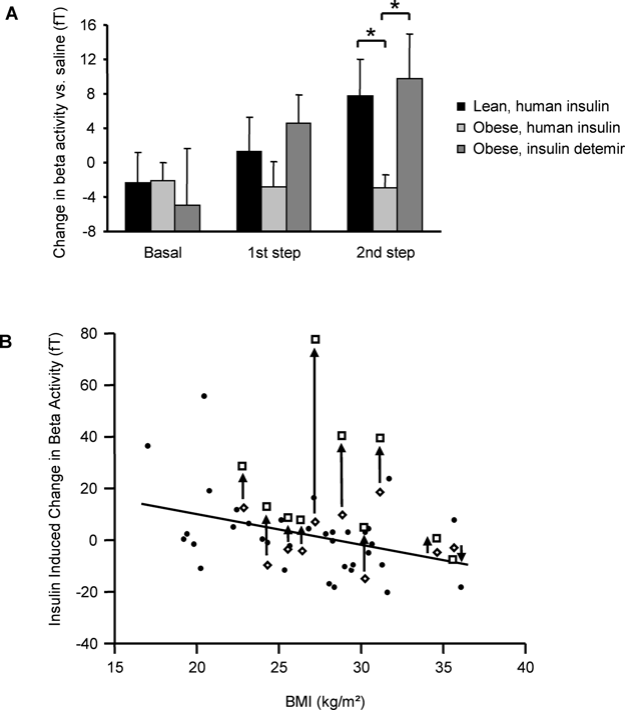
Insulin effects on beta activity in lean and overweight subjects. A: Comparison of the insulin detemir effect on beta activity with the effects human insulin in lean and overweight subjects. The figure shows beta activity from human insulin experiments in 12 lean and 34 overweight subjects and from insulin detemir experiments in 10 overweight subjects. Data from corresponding saline experiments have been subtracted. In the second step of the clamps beta activity was significantly higher in lean than in overweight subjects (p = 0.031) and higher with insulin detemir than with human insulin in overweight subjects (p = 0.040). B: Relationship between body-mass-index (BMI) and cerebral effects of insulin. The change in beta activity induced by human insulin (filled circles and open rhombs) was negatively correlated with BMI (r = −0.38, p = 0.0086) under hyperinsulinemic euglycemic clamped conditions as previously published [15]. The open rhombs represent the data obtained from the insulin experiment of 10 overweight subjects who additionally participated in the insulin detemir experiment. The change in beta activity induced by insulin detemir infusion is shown as open squares. An arrow indicates the corresponding values of each subject and illustrates the enhancement of cerebrocortical action of insulin detemir compared to human insulin. To account for lower glucose disposal in the insulin detemir experiments, the effect of insulin detemir on beta activity has been multiplied with the individual ratio of the glucose infusion rates (GIR_human insulin_/GIR_insulin detemir_).

To compare the effect of insulin detemir on beta activity with the effect of human insulin, we subtracted the beta activity measured during the saline experiment from beta activity measured during the human insulin experiment or the insulin detemir experiment in the same subject. At baseline, there was no difference between lean and overweight subjects studied with human insulin or overweight subjects studied with insulin detemir (all p>0.6). During the second step of infusion beta activity was increased in the lean group with human insulin compared to overweight individuals (p = 0.031) and with insulin detemir compared to human insulin in the overweight group (p = 0.040), while there was no difference between the human insulin effect in lean and the insulin detemir effect in obese subjects (p = 0.78) (Figure 3A).

In order to review the effect of insulin detemir in the context of the biological variation of cerebrocortical insulin effects, we show all data together in figure 3B. In this figure, the change of beta activity by human insulin is shown as filled circles and open rhombs. The open rhombs represent subjects who additionally participated in the insulin detemir experiment. First of all, the effect of human insulin on beta activity negatively correlates with BMI (r = −0.38, p = 0.0086) as previously published [15]. To further relate the impact of insulin detemir on the change of beta activity to the data obtained with human insulin, we added the data from the insulin detemir experiments in figure 3 B (open squares) and connected the corresponding data points of the human insulin and the insulin detemir experiment of each subject with an arrow. The figure shows that the effect of insulin detemir on beta activity is enhanced compared to human insulin in all subjects, except for the most obese one. In order to stress most specifically the difference of both insulins in stimulating beta activity, we “corrected” the effect of insulin detemir for the lower glucose infusion rates during insulin detemir experiments. For this purpose, the detemir effect on beta activity was multiplied with the ratio of glucose infusion rates (GIR_human insulin_/GIR_insulin detemir_) in each subject individually.

## Discussion

Insulin receptors in the brain play an important role in a variety of physiologic functions (memory, cognitive function, control of appetite, energy homeostasis, and endogenous glucose production). Cerebral insulin receptors seem to be involved in neurodegenerative diseases such as Alzheimer's disease and metabolic diseases such as obesity and type 2 diabetes. The prevalence of these diseases increases [23], [24] and, especially for obesity and diabetes, has reached epidemic proportions in western populations. Cerebral insulin resistance (i.e. reduced availability and/or effectiveness of insulin in the brain) might contribute to the pathogenesis of these diseases and, therefore, strategies to improve or overcome cerebral insulin resistance may become relevant for the therapy and prevention of obesity, type 2 diabetes and neurodegenerative diseases.

In a previous study, in which we established the detection of insulin effects on cerebrocortical activity with MEG, we have shown that human obesity is characterized by a reduced cerebral insulin response [15]. In the current study we designed a hyperinsulinemic euglycemic clamp with i.v. application of insulin detemir. As we hypothesized increased cerebrocortical action of the insulin analogue, it was necessary to achieve a similar or slightly lower effect on peripheral glucose metabolism than in the experiment using human insulin. Furthermore the time-profile of the plasma insulin concentrations had to be mimicked. First, 10 overweight subjects were investigated, and consistent with our previous findings human insulin did not change cortical activity. However, insulin detemir increased beta activity considerably, and therefore seems to improve the cerebrocortical response in beta activity. It is of note that the increase of the cerebrocortical effect was achieved despite lower peripheral effects, which justifies the conclusion of a brain-specific effect of insulin detemir in humans. Furthermore, the effect of insulin detemir in these overweight subjects was comparable to the effect of human insulin in the lean.

As proposed in the mouse study [19], an increased effect of insulin detemir in the brain may be explained by differences in albumin binding. In the brain, albumin concentrations are 200-fold lower than in the circulation [25], while in skeletal muscle they are only 5-fold lower [26]. In contrast to the brain, in skeletal muscle a considerable proportion of insulin detemir is bound to interstitial albumin. While in the circulation the albumin-bound insulin detemir appears to be metabolically inactive, it is unclear whether the local albumin concentrations in the skeletal muscle further reduces binding of insulin detemir to the receptor. In dogs, human insulin and insulin detemir induced a similar glucose uptake when interstitial concentrations of both insulins were similar, while the serum concentrations of insulin detemir were much higher than those of human insulin [27]. This finding indicates that in the circulation albumin-bound insulin detemir is metabolically inactive because it does not contribute to the passive transport to the interstitial fluid of the skeletal muscle while albumin-bound insulin detemir in the interstitial fluid contributes to receptor binding and is metabolically active. The main reason for the increased effect of insulin detemir in the brain is probably an increased transport across the blood-brain barrier. Insulin crosses the blood-brain barrier via an insulin receptor mediated active transport which is located on the vascular endothelium of brain blood vessels [28]. Like the insulin receptor in the skeletal muscle cell, this receptor is exposed to free and albumin-bound insulin detemir, however, in a much higher concentration (up to 40-fold higher). In contrast to the passive transport in peripheral tissues, the albumin-bound insulin detemir contributes to the active transport across the blood-brain barrier which leads to higher brain tissue concentrations as observed in mice [19].

Beta activity and other frequency bands are very unspecific measures of cerebrocortical activity. Changes in this parameter may reflect multiple functions and at the current stage no specific function can be assigned to the insulin effect. Therefore, it is unclear whether the increase of beta activity by insulin in lean subjects or by insulin detemir in overweight subjects is directly involved in body weight regulation, glucose tolerance or neuroprotection and whether it reflects a beneficial effect on brain function. However, we have some circumstantial evidence of a functional link as we recently found that a polymorphism in the *FTO* gene, which is related to obesity, is associated with a decreased insulin effect on cerebrocortical beta activity [29]. Though FTO is expressed in the brain, its function in humans is unclear. However, a decreased insulin response of the brain beta activity may contribute to the obesity effect of variation in this gene locus. Another interesting aspect is that insulin detemir has favorable effects on body weight development. Throughout all phase III studies, patients receiving insulin detemir displayed no weight gain or even weight loss, while patients receiving NPH insulin displayed weight gain [30]. This difference was observed under comparable glycemic control, strengthening the assumption of a specific weight-lowering effect of insulin detemir. Therefore, one may speculate that the increased cerebrocortical effect of insulin detemir in presence of comparable peripheral effects might be involved in the beneficial effects of insulin detemir on body weight development during insulin treatment.

In conclusion, we demonstrate that insulin detemir acts in the human brain more efficiently than human insulin at comparable or even lower peripheral metabolic effects. This tissue selectivity has already been demonstrated in mice and might be explained by the pharmacokinetic properties of insulin detemir. In the present study we could stimulate cerebrocortical beta activity in subjects who displayed no effect of human insulin in the brain. Therefore, insulin detemir seems to be a tool to restore at least in part the cerebrocortical insulin response in overweight humans. This principle may become a new therapeutic paradigm in obesity, type 2 diabetes and neurodegenerative diseases and might be applicable to other peptides which act in peripheral tissues and the brain.

## References

[1] Schwartz MW, Bergman RN, Kahn SE, Taborsky GJ, Fisher LD (1991). Evidence for entry of plasma insulin into cerebrospinal fluid through an intermediate compartment in dogs. Quantitative aspects and implications for transport.. J Clin Invest.

[2] Baura GD, Foster DM, Porte D, Kahn SE, Bergman RN (1993). Saturable transport of insulin from plasma into the central nervous system of dogs in vivo. A mechanism for regulated insulin delivery to the brain.. J Clin Invest.

[3] Woods SC, Lotter EC, McKay LD, Porte D (1979). Chronic intracerebroventricular infusion of insulin reduces food intake and body weight of baboons.. Nature.

[4] Porte D, Seeley RJ, Woods SC, Baskin DG, Figlewicz DP (1998). Obesity, diabetes and the central nervous system.. Diabetologia.

[5] Schwartz MW, Woods SC, Porte D, Seeley RJ, Baskin DG (2000). Central nervous system control of food intake.. Nature.

[6] Bruning JC, Gautam D, Burks DJ, Gillette J, Schubert M (2000). Role of brain insulin receptor in control of body weight and reproduction.. Science.

[7] Obici S, Zhang BB, Karkanias G, Rossetti L (2002). Hypothalamic insulin signaling is required for inhibition of glucose production.. Nat Med.

[8] Pocai A, Lam TK, Gutierrez-Juarez R, Obici S, Schwartz GJ (2005). Hypothalamic K(ATP) channels control hepatic glucose production.. Nature.

[9] Okamoto H, Obici S, Accili D, Rossetti L (2005). Restoration of liver insulin signaling in Insr knockout mice fails to normalize hepatic insulin action.. J Clin Invest.

[10] Biessels GJ, Kamal A, Urban IJ, Spruijt BM, Erkelens DW (1998). Water maze learning and hippocampal synaptic plasticity in streptozotocin-diabetic rats: effects of insulin treatment.. Brain Res.

[11] Born J, Lange T, Kern W, McGregor GP, Bickel U (2002). Sniffing neuropeptides: a transnasal approach to the human brain.. Nat Neurosci.

[12] Benedict C, Hallschmid M, Hatke A, Schultes B, Fehm HL (2004). Intranasal insulin improves memory in humans.. Psychoneuroendocrinology.

[13] Pilcher H (2006). Alzheimer's disease could be “type 3 diabetes”.. Lancet Neurol.

[14] Messier C, Teutenberg K (2005). The role of insulin, insulin growth factor, and insulin-degrading enzyme in brain aging and Alzheimer's disease.. Neural Plast.

[15] Tschritter O, Preissl H, Hennige AM, Stumvoll M, Porubska K (2006). The cerebrocortical response to hyperinsulinemia is reduced in overweight humans: A magnetoencephalographic study.. Proc Natl Acad Sci U S A.

[16] Havelund S, Plum A, Ribel U, Jonassen I, Volund A (2004). The mechanism of protraction of insulin detemir, a long-acting, acylated analog of human insulin.. Pharm Res.

[17] Kurtzhals P (2004). Engineering predictability and protraction in a basal insulin analogue: the pharmacology of insulin detemir.. Int J Obes Relat Metab Disord.

[18] Kaiyala KJ, Prigeon RL, Kahn SE, Woods SC, Schwartz MW (2000). Obesity induced by a high-fat diet is associated with reduced brain insulin transport in dogs.. Diabetes.

[19] Hennige AM, Sartorius T, Tschritter O, Preissl H, Fritsche A (2006). Tissue selectivity of insulin detemir action in vivo.. Diabetologia.

[20] Novitski N, Tervaniemi M, Huotilainen M, Naatanen R (2004). Frequency discrimination at different frequency levels as indexed by electrophysiological and behavioral measures.. Brain Res Cogn Brain Res.

[21] Sauseng P, Klimesch W, Doppelmayr M, Hanslmayr S, Schabus M (2004). Theta coupling in the human electroencephalogram during a working memory task.. Neurosci Lett.

[22] Lutzenberger W, Ripper B, Busse L, Birbaumer N, Kaiser J (2002). Dynamics of gamma-band activity during an audiospatial working memory task in humans.. J Neurosci.

[23] Mokdad AH, Ford ES, Bowman BA, Dietz WH, Vinicor F (2003). Prevalence of obesity, diabetes, and obesity-related health risk factors, 2001.. JAMA.

[24] Vardy ER, Hussain I, Hooper NM (2006). Emerging therapeutics for Alzheimer's disease.. Expert Rev Neurother.

[25] Seyfert S, Kunzmann V, Schwertfeger N, Koch HC, Faulstich A (2002). Determinants of lumbar CSF protein concentration.. J Neurol.

[26] Ellmerer M, Schaupp L, Brunner GA, Sendlhofer G, Wutte A (2000). Measurement of interstitial albumin in human skeletal muscle and adipose tissue by open-flow microperfusion.. Am J Physiol Endocrinol Metab.

[27] Dea MK, Hamilton-Wessler M, Ader M, Moore D, Schaffer L (2002). Albumin binding of acylated insulin (NN304) does not deter action to stimulate glucose uptake.. Diabetes.

[28] Woods SC, Seeley RJ, Baskin DG, Schwartz MW (2003). Insulin and the blood-brain barrier.. Curr Pharm Des.

[29] Tschritter O, Preissl H, Yokoyama Y, Machicao F, Haring HU (2007). Variation in the FTO gene locus is associated with cerebrocortical insulin resistance in humans.. Diabetologia. In press..

[30] Fritsche A, Haring H (2004). At last, a weight neutral insulin?. Int J Obes Relat Metab Disord.

